# (*E*)-Ethyl *N*′-(3,4-dimethoxy­benzyl­idene)hydrazinecarboxyl­ate monohydrate

**DOI:** 10.1107/S1600536808034417

**Published:** 2008-10-25

**Authors:** Lu-Ping Lv, Yong-Zhao Zhang, Xiao-Min Ding, Wei-Wei Li, Xian-Chao Hu

**Affiliations:** a Department of Chemical Engineering, Hangzhou Vocational and Technical College, Hangzhou 310018, People’s Republic of China; b Research Center of Analysis and Measurement, Zhejiang University of Technology, Hangzhou 310014, People’s Republic of China

## Abstract

In the title compound, C_12_H_16_N_2_O_4_·H_2_O, the mol­ecular skeleton of the hydrazinecarboxyl­ate is nearly planar [within 0.053 (3) Å]. In the crystal, chains propagating along the *c* axis arise, composed of alternating hydrazinecarboxyl­ate mol­ecules and crystalline water, which inter­act *via* N—H⋯O and O—H⋯O hydrogen bonds.

## Related literature

For general background, see: Parashar *et al.* (1988 ▶); Hadjoudis *et al.* (1987 ▶); Borg *et al.* (1999 ▶). For a related structure, see Shang *et al.* (2007 ▶).
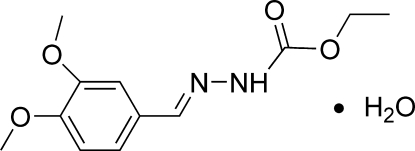


## Experimental

### 

#### Crystal data

C_12_H_16_N_2_O_4_·H_2_O
*M* *_r_* = 270.28
Orthorhombic, 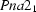

*a* = 7.211 (2) Å
*b* = 17.688 (5) Å
*c* = 11.026 (3) Å
*V* = 1406.3 (7) Å^3^
*Z* = 4
Mo *K*α radiation
μ = 0.10 mm^−1^
*T* = 193 (2) K
0.27 × 0.25 × 0.23 mm

#### Data collection

Bruker SMART CCD area-detector diffractometer
Absorption correction: multi-scan (*SADABS*; Bruker, 2002 ▶) *T*_min_ = 0.969, *T*_max_ = 0.976
7281 measured reflections
1312 independent reflections
1181 reflections with *I* > 2σ(*I*)
*R*_int_ = 0.026

#### Refinement

*R*[*F* ^2^ > 2σ(*F* ^2^)] = 0.031
*wR*(*F* ^2^) = 0.085
*S* = 1.07
1312 reflections
180 parameters
3 restraints
H atoms treated by a mixture of independent and constrained refinement
Δρ_max_ = 0.15 e Å^−3^
Δρ_min_ = −0.15 e Å^−3^

### 

Data collection: *SMART* (Bruker, 2002 ▶); cell refinement: *SAINT* (Bruker, 2002 ▶); data reduction: *SAINT*; program(s) used to solve structure: *SHELXS97* (Sheldrick, 2008 ▶); program(s) used to refine structure: *SHELXL97* (Sheldrick, 2008 ▶); molecular graphics: *SHELXTL* (Sheldrick, 2008 ▶); software used to prepare material for publication: *SHELXTL*.

## Supplementary Material

Crystal structure: contains datablocks I, global. DOI: 10.1107/S1600536808034417/cv2464sup1.cif

Structure factors: contains datablocks I. DOI: 10.1107/S1600536808034417/cv2464Isup2.hkl

Additional supplementary materials:  crystallographic information; 3D view; checkCIF report

## Figures and Tables

**Table 1:** Hydrogen-bond geometry (Å, °)

*D*—H⋯*A*	*D*—H	H⋯*A*	*D*⋯*A*	*D*—H⋯*A*
O1*W*—H1*F*⋯O3	0.833 (19)	2.14 (2)	2.899 (3)	151 (3)
N2—H2⋯O1*W*^i^	0.88	2.12	2.899 (3)	148

Symmetry code: (i) 
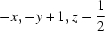
.

**Fig. 1.**
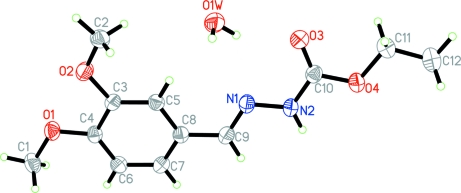
Molecular structure of (I), showing 30% probability displacement ellipsoids and the atomic numbering.

## supplementary crystallographic information

### Comment

Benzaldehydehydrazone derivatives have received considerable attention for a long time due to their pharmacological activity (Parashar *et al.*,  1988) and photochromic properties (Hadjoudis *et al.*,  1987). Meanwhile, it is an important intermediate of 1,3,4-oxadiazoles, which have been reported to be compounds with  versatile useful properties (Borg *et al.*,  1999). As a further investigation of this type of derivatives, we report herein the crystal structure of the title compound.

In the title compound, C_12_H_16_N_2_O_4_ (I) .H_2_O, the molecular skeleton of (I) is nearly planar. The main molecule, (I), adopts a trans configuration with respect to the C═N bond. The hydrazine carboxylic acid methyl ester group is slightly twisted away from the attached ring. The bond lengths and angles of the main molecule agree with those observed in (E)-methyl N'-(4-hydroxybenzylidene)hydrazinecarboxylate (Shang *et al.*, 2007).

The crystal packing exhibits hydrogen-bonded chains extended along c axis and composed from alternating molecules of (I) and crystalline water, which interact via N-H···O [N···O 2.899 (3) Å] and O-H···O [O···O 2.899 (3) Å] hydrogen bonds (Table 1).

### Experimental

3,4-Dimethoxybenzaldehyde (1.66g, 0.01mol) and ethyl hydrazinecarboxylate(1.04g, 0.01mol) were dissolved in stirred methanol (20ml) and left for 3h at room temperature. The resulting solid was filtered off and recrystallized from ethanol to give the title compound in 90% yield.  Crystals suitable for X-ray analysis were obtained by slow evaporation of a ethanol solution at room temperature (m.p. 458-460 K).

### Refinement

H atoms of the water molecule were located in a difference map and were refined with O-H distances restrained to 0.82 (2) Å, H atoms were included in the riding model approximation with N-H = 0.88 Å. C-bound H atoms were positioned geometrically (C-H = 0.95-0.99 Å) and refined using a riding model, with U_iso_(H) = 1.2-1.5U_eq_(C). In the absence of significant anomalous scatterers, the 1193 Friedel pairs were merged before the final refinement.

### Crystal data

**Table:**

C_12_H_16_N_2_O_4_·H_2_O	*F*(000) = 576
*M**_r_* = 270.28	*D*_x_ = 1.277 Mg m^−^^3^
Orthorhombic, *P**n**a*2_1_	Mo *K*α radiation, λ = 0.71073 Å
Hall symbol: P 2c -2n	Cell parameters from 1312 reflections
*a* = 7.211 (2) Å	θ = 2.2–25.5°
*b* = 17.688 (5) Å	µ = 0.10 mm^−^^1^
*c* = 11.026 (3) Å	*T* = 193 K
*V* = 1406.3 (7) Å^3^	Block, colourless
*Z* = 4	0.27 × 0.25 × 0.23 mm

### Data collection

**Table:**

Bruker SMART CCD area-detector diffractometer	1312 independent reflections
Radiation source: fine-focus sealed tube	1181 reflections with *I* > 2σ(*I*)
graphite	*R*_int_ = 0.026
φ and ω scans	θ_max_ = 25.0°, θ_min_ = 2.2°
Absorption correction: multi-scan (SADABS; Bruker, 2002)	*h* = −8→8
*T*_min_ = 0.969, *T*_max_ = 0.976	*k* = −20→20
7281 measured reflections	*l* = −13→12

### Refinement

**Table:**

Refinement on *F*^2^	Primary atom site location: structure-invariant direct methods
Least-squares matrix: full	Secondary atom site location: difference Fourier map
*R*[*F*^2^ > 2σ(*F*^2^)] = 0.031	Hydrogen site location: inferred from neighbouring sites
*wR*(*F*^2^) = 0.085	H atoms treated by a mixture of independent and constrained refinement
*S* = 1.07	*w* = 1/[σ^2^(*F*_o_^2^) + (0.0551*P*)^2^ + 0.0753*P*] where *P* = (*F*_o_^2^ + 2*F*_c_^2^)/3
1312 reflections	(Δ/σ)_max_ < 0.001
180 parameters	Δρ_max_ = 0.16 e Å^−^^3^
3 restraints	Δρ_min_ = −0.15 e Å^−^^3^

### Special details

**Table:**

Geometry. All esds (except the esd in the dihedral angle between two l.s. planes) are estimated using the full covariance matrix. The cell esds are taken into account individually in the estimation of esds in distances, angles and torsion angles; correlations between esds in cell parameters are only used when they are defined by crystal symmetry. An approximate (isotropic) treatment of cell esds is used for estimating esds involving l.s. planes.
Refinement. Refinement of F^2^ against ALL reflections. The weighted R-factor wR and goodness of fit S are based on F^2^, conventional R-factors R are based on F, with F set to zero for negative F^2^. The threshold expression of F^2^ > 2sigma(F^2^) is used only for calculating R-factors(gt) etc. and is not relevant to the choice of reflections for refinement. R-factors based on F^2^ are statistically about twice as large as those based on F, and R- factors based on ALL data will be even larger.

### Fractional atomic coordinates and isotropic or equivalent isotropic displacement parameters (Å^2^)

**Table:**

	*x*	*y*	*z*	*U*_iso_*/*U*_eq_	
C1	1.0427 (4)	0.67958 (16)	0.2893 (3)	0.0656 (7)	
H1A	1.1372	0.7154	0.3172	0.098*	
H1B	0.9915	0.6969	0.2117	0.098*	
H1C	1.0989	0.6296	0.2786	0.098*	
C2	0.4765 (4)	0.69467 (15)	0.6084 (3)	0.0610 (6)	
H2A	0.5117	0.7319	0.6701	0.091*	
H2B	0.4388	0.6476	0.6482	0.091*	
H2C	0.3730	0.7144	0.5603	0.091*	
C3	0.6065 (3)	0.62962 (12)	0.4381 (2)	0.0458 (5)	
C4	0.7529 (3)	0.62623 (13)	0.3535 (2)	0.0458 (6)	
C5	0.4527 (3)	0.58339 (12)	0.4241 (2)	0.0462 (5)	
H5	0.3536	0.5859	0.4806	0.055*	
C6	0.7444 (3)	0.57723 (14)	0.2568 (2)	0.0514 (6)	
H6	0.8428	0.5753	0.1995	0.062*	
C7	0.5889 (3)	0.53002 (14)	0.2433 (2)	0.0519 (6)	
H7	0.5833	0.4959	0.1769	0.062*	
C8	0.4438 (3)	0.53255 (13)	0.3255 (2)	0.0469 (5)	
C9	0.2842 (3)	0.48227 (14)	0.3094 (3)	0.0513 (6)	
H9	0.2729	0.4534	0.2371	0.062*	
C10	−0.1176 (4)	0.42102 (13)	0.4535 (2)	0.0511 (6)	
C11	−0.4016 (4)	0.36234 (19)	0.5028 (3)	0.0719 (8)	
H11A	−0.4699	0.4105	0.5130	0.086*	
H11B	−0.3517	0.3471	0.5828	0.086*	
C12	−0.5286 (5)	0.3030 (2)	0.4560 (4)	0.0882 (11)	
H12A	−0.6307	0.2956	0.5134	0.132*	
H12B	−0.4602	0.2555	0.4463	0.132*	
H12C	−0.5786	0.3188	0.3773	0.132*	
N1	0.1595 (3)	0.47690 (10)	0.3921 (2)	0.0520 (5)	
N2	0.0153 (3)	0.42736 (11)	0.3681 (2)	0.0525 (5)	
H2	0.0107	0.4012	0.3002	0.063*	
O1	0.8976 (2)	0.67502 (9)	0.37704 (18)	0.0578 (5)	
O2	0.6305 (2)	0.68036 (11)	0.53107 (17)	0.0620 (5)	
O3	−0.1179 (3)	0.45274 (11)	0.55163 (18)	0.0689 (5)	
O4	−0.2508 (2)	0.37243 (9)	0.41722 (18)	0.0582 (5)	
O1W	0.0397 (3)	0.59890 (12)	0.6111 (2)	0.0667 (5)	
H1E	0.003 (5)	0.6288 (18)	0.561 (3)	0.090 (12)*	
H1F	0.030 (5)	0.5563 (13)	0.579 (3)	0.088 (12)*	

### Atomic displacement parameters (Å^2^)

**Table:**

	*U*^11^	*U*^22^	*U*^33^	*U*^12^	*U*^13^	*U*^23^
C1	0.0528 (15)	0.0662 (16)	0.0777 (18)	−0.0115 (12)	0.0121 (14)	−0.0002 (15)
C2	0.0693 (16)	0.0566 (14)	0.0570 (15)	−0.0026 (12)	0.0121 (15)	−0.0057 (13)
C3	0.0457 (12)	0.0442 (11)	0.0474 (12)	0.0005 (8)	−0.0055 (11)	−0.0020 (10)
C4	0.0389 (12)	0.0447 (10)	0.0537 (15)	0.0004 (9)	−0.0033 (10)	0.0030 (11)
C5	0.0414 (11)	0.0506 (12)	0.0466 (13)	−0.0001 (9)	−0.0005 (10)	0.0029 (10)
C6	0.0456 (12)	0.0538 (12)	0.0548 (15)	0.0026 (10)	0.0029 (11)	0.0000 (12)
C7	0.0536 (13)	0.0510 (12)	0.0513 (13)	0.0004 (10)	−0.0048 (12)	−0.0061 (11)
C8	0.0430 (12)	0.0462 (11)	0.0515 (13)	0.0010 (9)	−0.0065 (11)	0.0051 (10)
C9	0.0512 (12)	0.0483 (12)	0.0543 (14)	−0.0020 (10)	−0.0075 (12)	−0.0013 (11)
C10	0.0553 (14)	0.0453 (11)	0.0526 (14)	−0.0034 (10)	−0.0067 (12)	0.0022 (11)
C11	0.0618 (17)	0.082 (2)	0.0724 (19)	−0.0143 (14)	0.0081 (15)	0.0022 (15)
C12	0.0713 (19)	0.097 (2)	0.096 (3)	−0.0260 (18)	0.004 (2)	0.003 (2)
N1	0.0493 (11)	0.0485 (10)	0.0582 (13)	−0.0066 (8)	−0.0074 (11)	0.0029 (9)
N2	0.0500 (11)	0.0521 (11)	0.0554 (13)	−0.0116 (8)	−0.0024 (10)	−0.0037 (9)
O1	0.0451 (9)	0.0621 (10)	0.0661 (11)	−0.0105 (7)	0.0053 (9)	−0.0080 (9)
O2	0.0529 (10)	0.0723 (10)	0.0607 (12)	−0.0111 (8)	0.0041 (9)	−0.0181 (10)
O3	0.0744 (12)	0.0770 (11)	0.0553 (11)	−0.0204 (10)	0.0011 (10)	−0.0043 (10)
O4	0.0546 (10)	0.0570 (9)	0.0628 (12)	−0.0138 (7)	0.0022 (8)	−0.0010 (9)
O1W	0.0778 (13)	0.0662 (12)	0.0561 (12)	−0.0123 (10)	−0.0078 (11)	0.0050 (11)

### Geometric parameters (Å, °)

**Table:**

C1—O1	1.427 (3)	C7—H7	0.9500
C1—H1A	0.9800	C8—C9	1.465 (3)
C1—H1B	0.9800	C9—N1	1.284 (3)
C1—H1C	0.9800	C9—H9	0.9500
C2—O2	1.423 (3)	C10—O3	1.219 (3)
C2—H2A	0.9800	C10—N2	1.348 (3)
C2—H2B	0.9800	C10—O4	1.350 (3)
C2—H2C	0.9800	C11—O4	1.451 (4)
C3—O2	1.374 (3)	C11—C12	1.485 (5)
C3—C5	1.387 (3)	C11—H11A	0.9900
C3—C4	1.410 (3)	C11—H11B	0.9900
C4—C6	1.376 (4)	C12—H12A	0.9800
C4—O1	1.379 (3)	C12—H12B	0.9800
C5—C8	1.412 (4)	C12—H12C	0.9800
C5—H5	0.9500	N1—N2	1.385 (3)
C6—C7	1.406 (4)	N2—H2	0.8800
C6—H6	0.9500	O1W—H1E	0.812 (19)
C7—C8	1.385 (3)	O1W—H1F	0.833 (19)
			
O1—C1—H1A	109.5	C7—C8—C9	119.6 (2)
O1—C1—H1B	109.5	C5—C8—C9	121.0 (2)
H1A—C1—H1B	109.5	N1—C9—C8	120.6 (2)
O1—C1—H1C	109.5	N1—C9—H9	119.7
H1A—C1—H1C	109.5	C8—C9—H9	119.7
H1B—C1—H1C	109.5	O3—C10—N2	125.7 (2)
O2—C2—H2A	109.5	O3—C10—O4	123.7 (2)
O2—C2—H2B	109.5	N2—C10—O4	110.6 (2)
H2A—C2—H2B	109.5	O4—C11—C12	108.8 (3)
O2—C2—H2C	109.5	O4—C11—H11A	109.9
H2A—C2—H2C	109.5	C12—C11—H11A	109.9
H2B—C2—H2C	109.5	O4—C11—H11B	109.9
O2—C3—C5	124.7 (2)	C12—C11—H11B	109.9
O2—C3—C4	115.30 (19)	H11A—C11—H11B	108.3
C5—C3—C4	120.0 (2)	C11—C12—H12A	109.5
C6—C4—O1	125.0 (2)	C11—C12—H12B	109.5
C6—C4—C3	120.4 (2)	H12A—C12—H12B	109.5
O1—C4—C3	114.5 (2)	C11—C12—H12C	109.5
C3—C5—C8	119.8 (2)	H12A—C12—H12C	109.5
C3—C5—H5	120.1	H12B—C12—H12C	109.5
C8—C5—H5	120.1	C9—N1—N2	115.9 (2)
C4—C6—C7	119.5 (2)	C10—N2—N1	116.9 (2)
C4—C6—H6	120.3	C10—N2—H2	121.5
C7—C6—H6	120.3	N1—N2—H2	121.5
C8—C7—C6	120.9 (2)	C4—O1—C1	117.5 (2)
C8—C7—H7	119.5	C3—O2—C2	117.7 (2)
C6—C7—H7	119.5	C10—O4—C11	114.8 (2)
C7—C8—C5	119.3 (2)	H1E—O1W—H1F	106 (4)
			
O2—C3—C4—C6	179.8 (2)	C7—C8—C9—N1	171.3 (2)
C5—C3—C4—C6	0.0 (3)	C5—C8—C9—N1	−8.3 (3)
O2—C3—C4—O1	−0.4 (3)	C8—C9—N1—N2	−179.5 (2)
C5—C3—C4—O1	179.8 (2)	O3—C10—N2—N1	−3.1 (4)
O2—C3—C5—C8	−179.2 (2)	O4—C10—N2—N1	178.61 (19)
C4—C3—C5—C8	0.5 (3)	C9—N1—N2—C10	−179.8 (2)
O1—C4—C6—C7	179.7 (2)	C6—C4—O1—C1	5.4 (3)
C3—C4—C6—C7	−0.5 (3)	C3—C4—O1—C1	−174.4 (2)
C4—C6—C7—C8	0.5 (4)	C5—C3—O2—C2	−9.6 (3)
C6—C7—C8—C5	0.0 (4)	C4—C3—O2—C2	170.6 (2)
C6—C7—C8—C9	−179.6 (2)	O3—C10—O4—C11	2.3 (3)
C3—C5—C8—C7	−0.5 (3)	N2—C10—O4—C11	−179.4 (2)
C3—C5—C8—C9	179.1 (2)	C12—C11—O4—C10	−175.3 (3)

### Hydrogen-bond geometry (Å, °)

**Table:**

*D*—H···*A*	*D*—H	H···*A*	*D*···*A*	*D*—H···*A*
O1W—H1F···O3	0.83 (2)	2.14 (2)	2.899 (3)	151 (3)
O1W—H1E···O1^i^	0.81 (2)	2.31 (2)	3.086 (3)	160 (4)
N2—H2···O1W^ii^	0.88	2.12	2.899 (3)	148

Symmetry codes: (i) *x*−1, *y*, *z*; (ii) −*x*, −*y*+1, *z*−1/2.
